# Supplementary figures and images for: Antibody-mediated depletion of select leukocyte subsets in blood and tissue of nonhuman primates
Source: Front Immunol. 2024 Mar 11;15:1359679. doi: 10.3389/fimmu.2024.1359679 (PMC10961357; doi:10.3389/fimmu.2024.1359679)

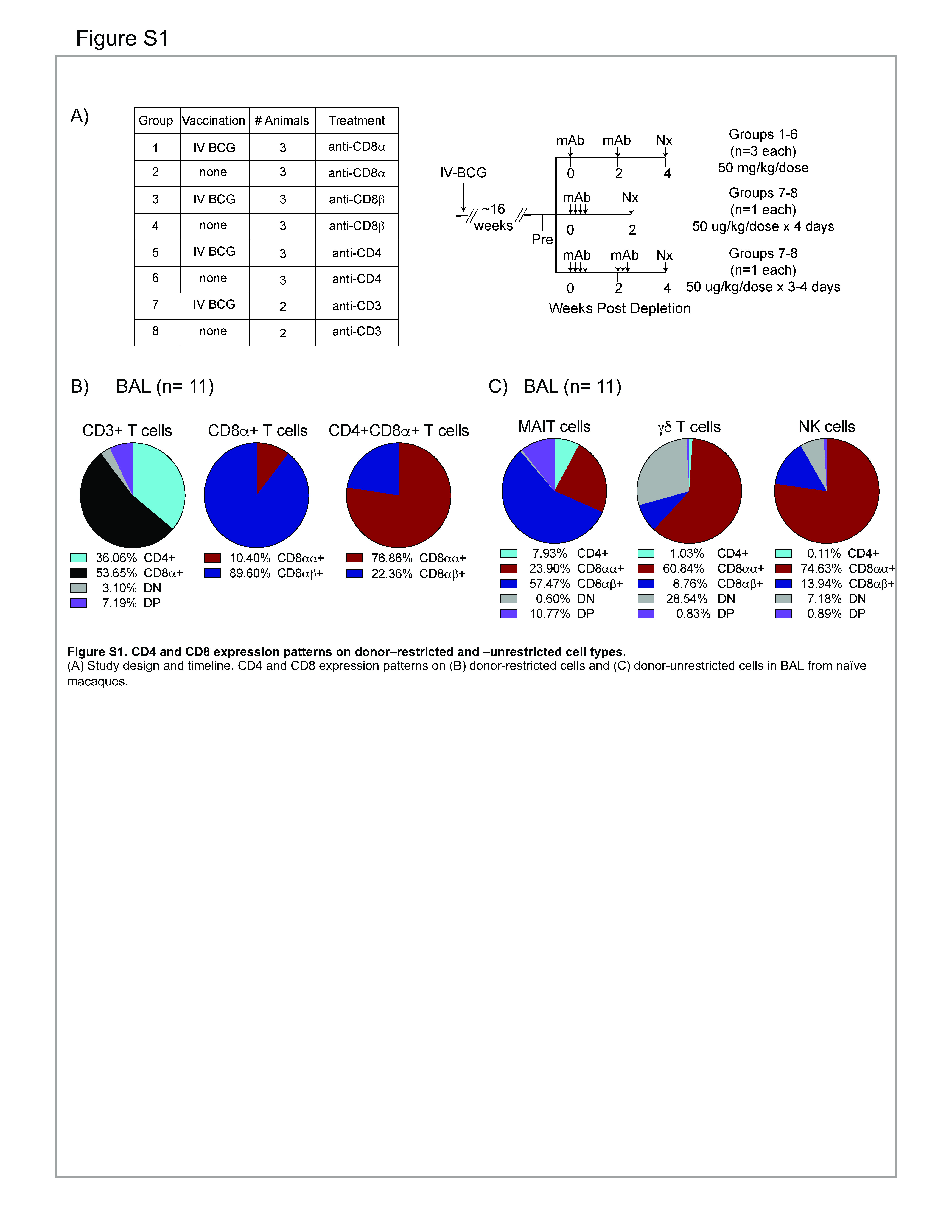

Supplement: Supplementary file 1 [file Image_1.jpg]

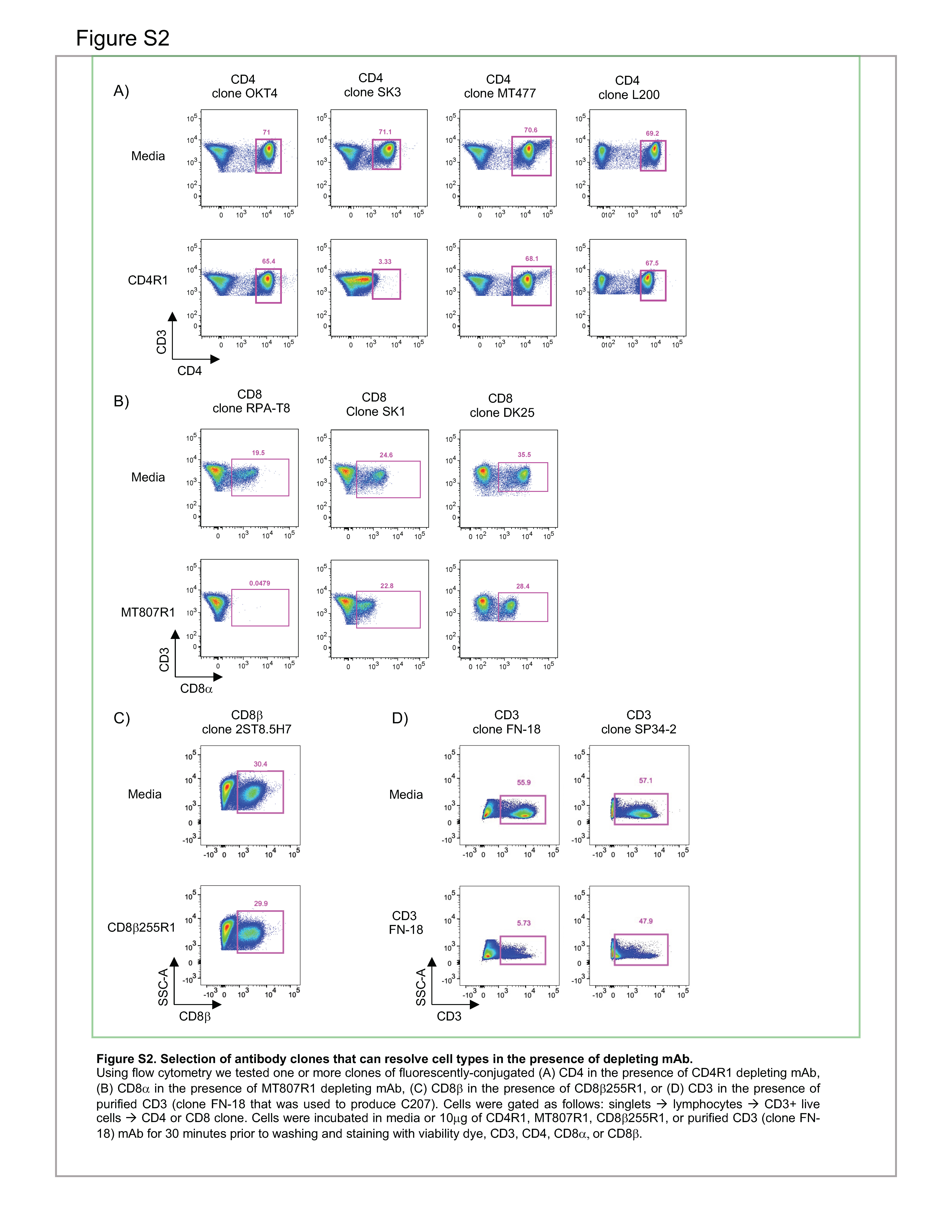

Supplement: Supplementary file 2 [file Image_2.jpg]

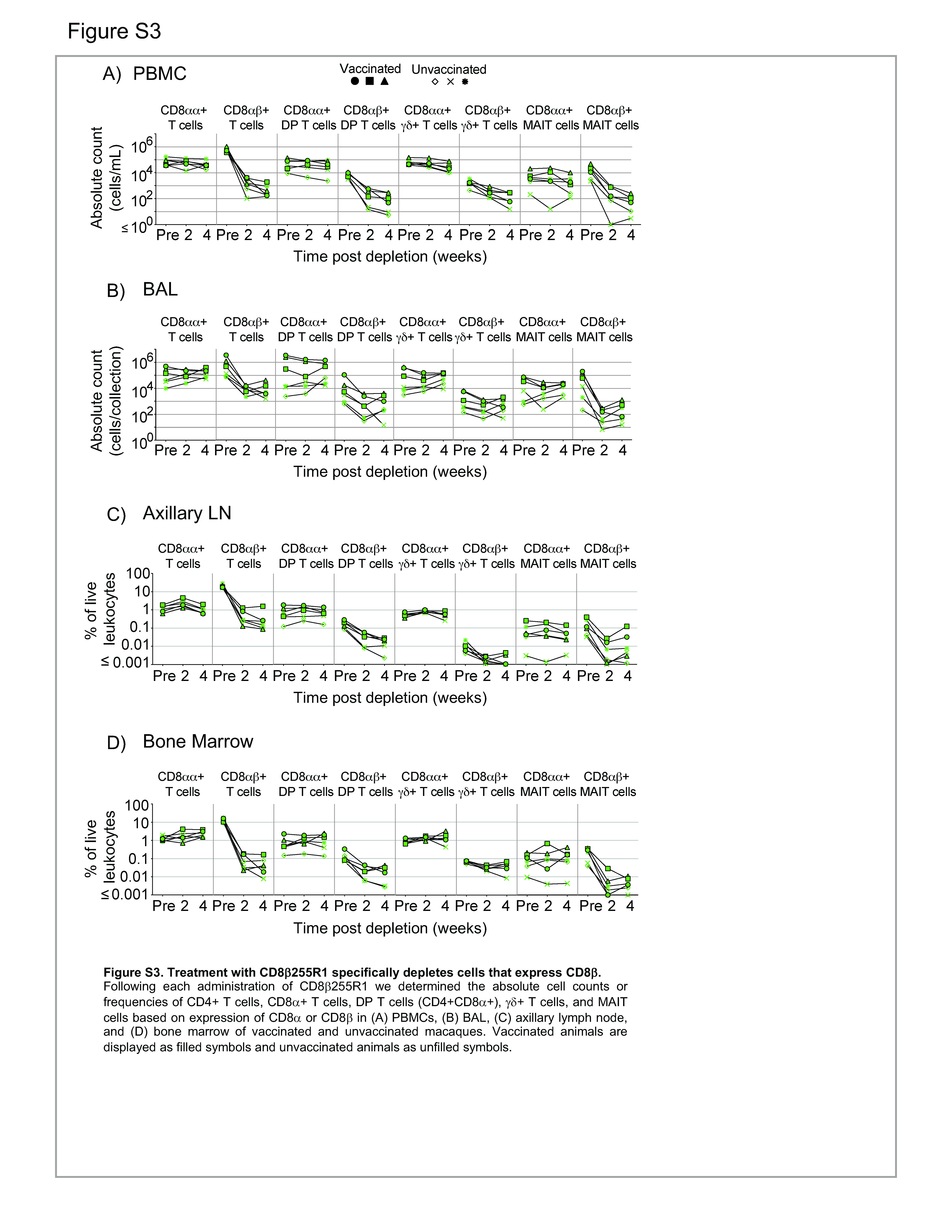

Supplement: Supplementary file 3 [file Image_3.jpg]

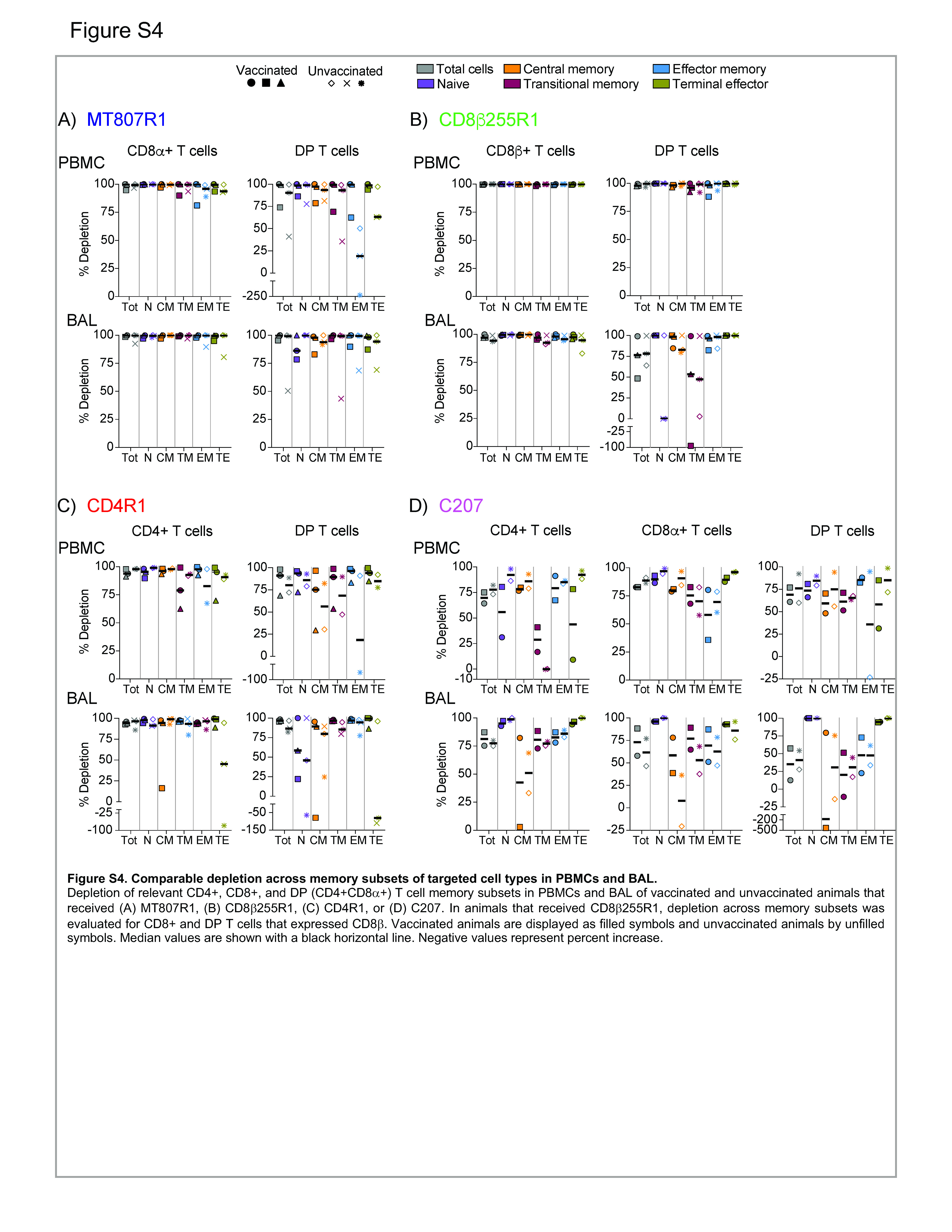

Supplement: Supplementary file 4 [file Image_4.jpg]

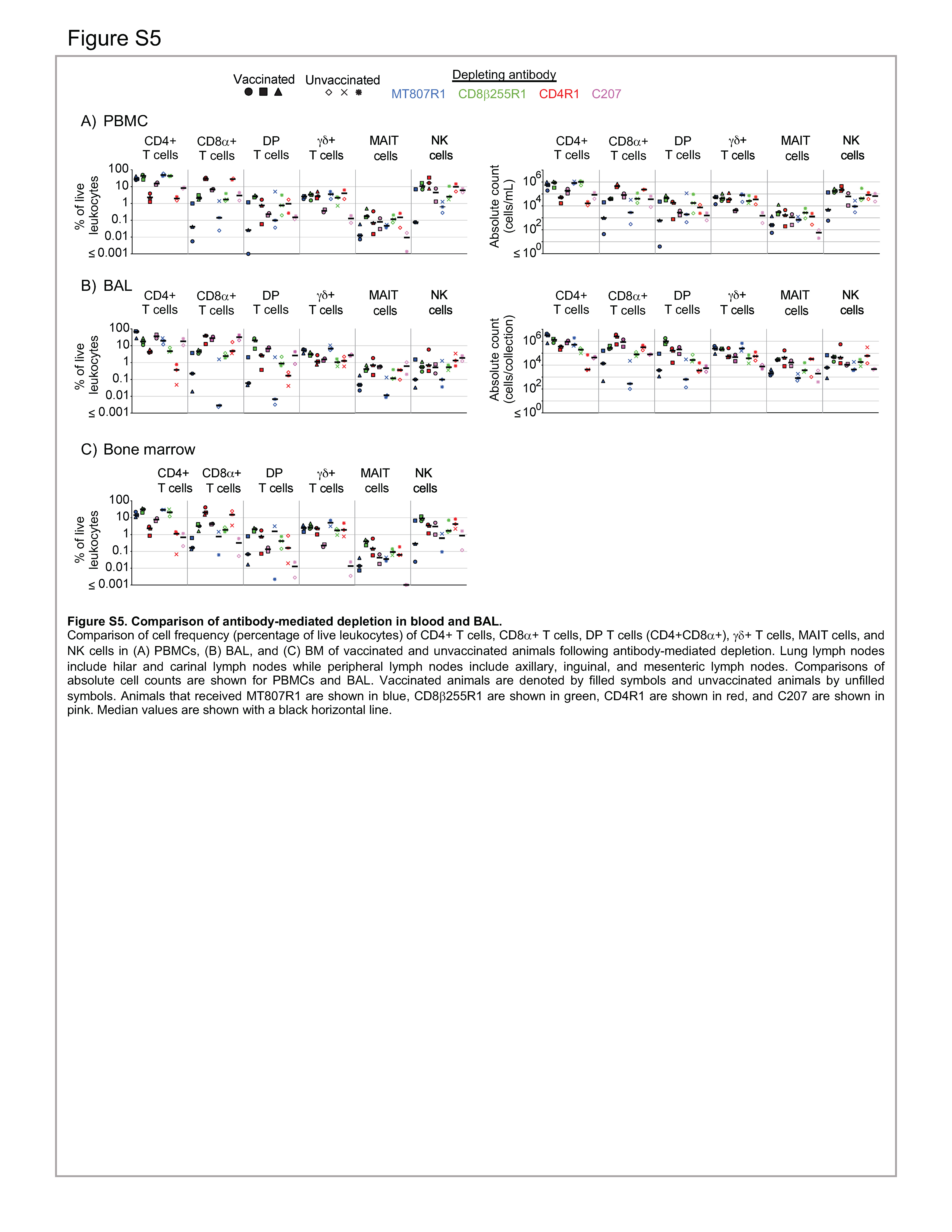

Supplement: Supplementary file 5 [file Image_5.jpg]

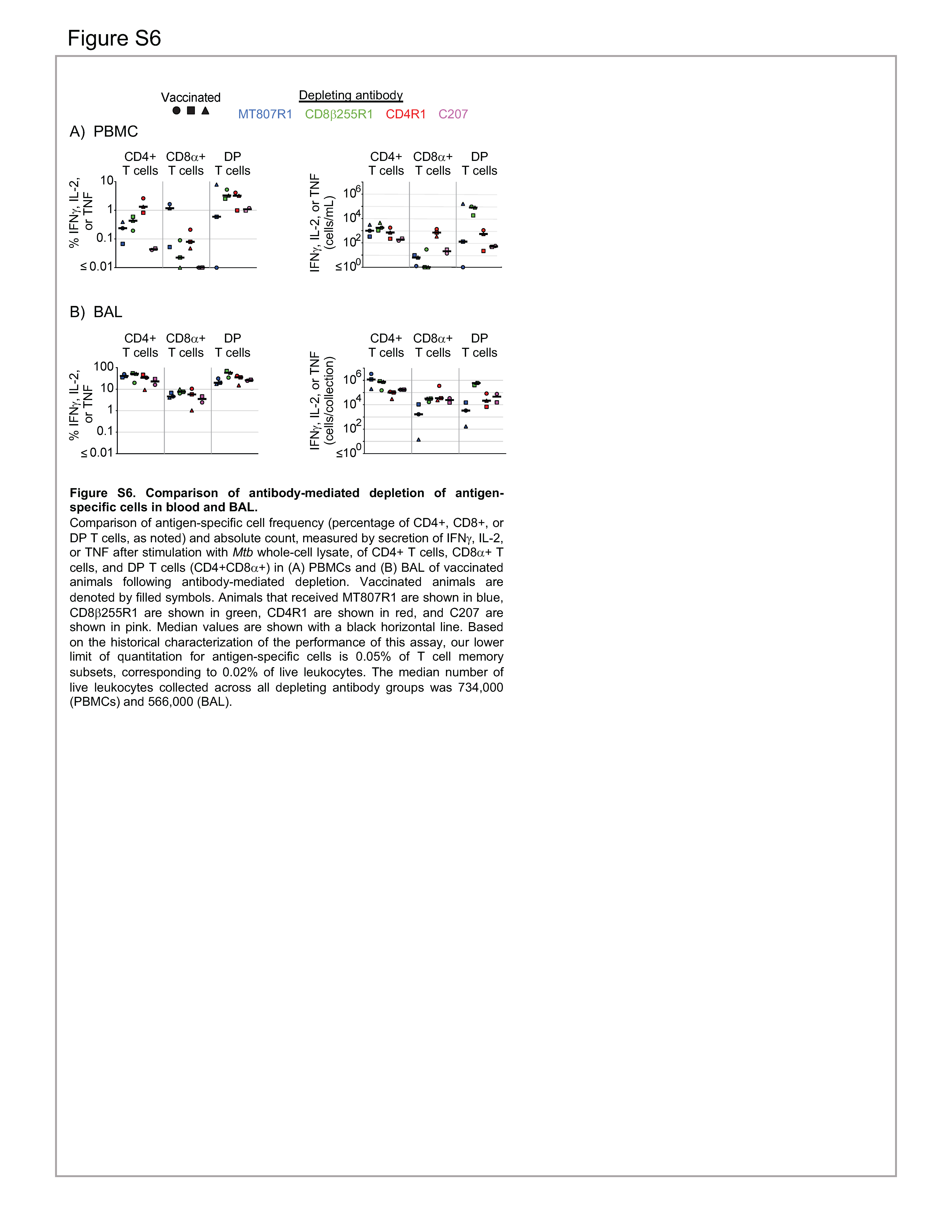

Supplement: Supplementary file 6 [file Image_6.jpg]
